# Burden of hospitalization in acute lymphoblastic leukemia patients treated with Inotuzumab Ozogamicin versus standard chemotherapy treatment

**DOI:** 10.1002/cam4.2480

**Published:** 2019-08-22

**Authors:** David I. Marks, Ilse van Oostrum, Sabrina Mueller, Verna Welch, Erik Vandendries, Fausto R. Loberiza, Sarah Böhme, Yun Su, Matthias Stelljes, Hagop M. Kantarjian

**Affiliations:** ^1^ Bristol Haematology and Oncology Centre University Hospitals Bristol NHS Foundation Trust Bristol UK; ^2^ Ingress‐Health Nederland B.V. Rotterdam The Netherlands; ^3^ Ingress‐Health HWM GmbH Wismar Germany; ^4^ Pfizer Global HEOR New York NY USA; ^5^ Pfizer Inc Cambridge MA USA; ^6^ Pfizer Inc, Pharma GmbH Berlin Germany; ^7^ Pfizer Deutschland GmbH Berlin Germany; ^8^ Independent Bridgewater NJ USA; ^9^ Universitätsklinikum Münster Münster Germany; ^10^ University of Texas MD Anderson Cancer Center Houston TX USA

**Keywords:** acute lymphoblastic leukemia, chemotherapy, hospitalization, inotuzumab ozogamicin

## Abstract

**Background:**

Inotuzumab Ozogamicin (INO), has demonstrated an improvement in overall survival, high rate of complete remission, favorable patient‐reported outcomes, and manageable safety profile vs standard of care (SoC; intensive chemotherapy) for relapsed/refractory (R/R) acute lymphoblastic leukemia (ALL) in the phase 3 INO‐VATE trial. With a one‐hour weekly dosing schedule, INO might be associated with lower healthcare system burden. This study analyses hospitalizations for INO vs SoC.

**Methods:**

All patients receiving study treatment in the INO‐VATE trial were included. The days hospitalized during study treatment was calculated. Due to different treatment durations for INO and SoC (median of 3 vs 1 cycles), number of hospital days was mainly reported per observed patient month. Hospital days per patient month were analyzed for different treatment cycles, subgroups, and main reasons for hospitalization. Differences between treatments were analyzed by the incidence rate ratio (IRR).

**Results:**

Overall, 82.9% and 94.4% INO and SoC patients experienced at least one hospitalization. The mean hospitalization days per patient month was 7.6 and 18.4 days for INO and SoC (IRR = 0.413, *P* < .001), which corresponds to patients spending 25.0% and 60.5% of their treatment time in a hospital. Main hospitalization reasons were R/R ALL treatment (5.2 (INO) vs 14.0 (SoC) days, IRR = 0.368, *P* < .001), treatment toxicities (1.4 vs 2.8 days, IRR = 0.516, *P* < .001) or other reasons (1.0 vs 1.6 days, IRR 0.629, *P* < .001).

**Conclusions:**

Inotuzumab Ozogamicin treatment in R/R ALL is associated with a lower hospitalization burden compared with SoC. It is likely this lower burden has a favorable impact on healthcare budgets and cost‐effectiveness considerations.

## Graphical abstract

Compared to current standard of care (intensive chemotherapy), Inotuzumab Ozogamicin reduces the hospitalization burden in relapsed/refractory acute lymphoblastic leukemia patients. It is likely this lower burden has a favorable impact on the quality of life of patients, healthcare budgets, and cost‐effectiveness considerations.

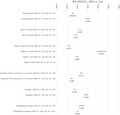

## 1 BACKGROUND AND OBJECTIVES

Acute lymphocytic leukemia (ALL) is a life‐threatening diagnosis.^1^ Current therapies for adults with newly diagnosed B‐cell ALL are associated with rates of complete remission (CR) as high as 60%‐90%.^2^, ^3^, ^4^, ^5^, ^6^, ^7^, ^8^, ^9^ However, many of the patients with CR experience a relapse.^2^, ^3^, ^4^, ^5^, ^6^, ^7^, ^8^, ^9^ For these patients the estimated 5‐year survival rate is less than 10%. The prognosis of adults with relapsed or refractory B‐cell ALL (R/R ALL) depends on several parameters, including response to prior salvage therapy, duration of first remission, patient age, and disease burden at time of relapse.^10^ The only curative option consists of achieving a second CR by salvage therapy followed by an allogenic hematopoietic stem cell transplantation (HSCT), but less than half of the patients achieve a second CR and only a limited subset of patients are eligible for this procedure.^6^, ^11^, ^12^, ^13^ Standard chemotherapy regimens for adults with R/R ALL are associated with rates of CR of 31% to 44% when they are the first salvage therapy administered after an early relapse, and 18%‐25% when they are the second salvage therapy.^10^, ^11^, ^14^, ^15^ So, as CR is generally considered a prerequisite for subsequent HSCT, these low rates of CR mean that few adults with R/R ALL proceed to HSCT; a potential curative option.

A Phase III trial confirmed that Inotuzumab Ozogamicin (INO), an anti‐CD22 antibody conjugated to calicheamicin, results in better outcomes in patients with R/R ALL than standard of care (SoC) chemotherapy, with a manageable safety profile. In the INO‐VATE ALL trial, INO was associated with higher rates of CR/CRi within the ITT218 population than SoC (80.7% vs 29.4%, *P* < .001). The estimated HR for the second primary endpoint of OS was 0.770 (97.5% CI, 0.578‐1.026), with one‐sided *P* = .0203 in favor of INO over control therapy based on the stratified analysis, indicating an overall 23% reduction in the risk of death in favor of INO. The survival probability at 24 months was 23% (95% CI, 16%‐30%) in the INO arm and 10% (5%‐16%) in the control arm.^16^

Treatment of R/R ALL is associated with a significant burden for both patients and health care systems, the latter mainly because of frequent and lengthy hospitalizations of patients. High rates of hospitalizations in this patient group can be explained by limited effectiveness of standard chemotherapy, potential toxicity of that treatment, and inconvenient chemotherapy dosing schedules.^17^ Several previous studies reported that R/R ALL patients undergoing chemotherapy spent about 50% of their treatment time, defined as time between first and last administration of a dosage, in hospital.^17^, ^18^, ^19^, ^20^

Due to its superior efficacy and manageable safety profile as well as a convenient one‐hour weekly dosing schedule,^16^ INO might be associated with lower health care system burden, especially because of lower hospitalization frequency during treatment periods. Data in this respect have not been published so far. That is why, the main objective of this study was to analyze hospitalization frequency of R/R ALL patients who received either INO or SoC chemotherapy, based on the data collected in the INO‐VATE ALL trial.

## 2 MATERIALS AND METHODS

The INO‐VATE ALL trial has already been described in detail elsewhere.^16^ In short, INO‐VATE ALL was an open‐label, randomized, controlled phase 3 trial on adult R/R ALL patients who were scheduled to receive their first or second salvage treatment. Patients were randomly assigned, in a 1:1 ratio, to receive either INO or SoC (investigator's choice); no crossover between groups was allowed. Patients who achieved complete remission could undergo stem‐cell transplantation at the investigator's discretion.

Patients in the INO group received the trial drug intravenously at a starting dose of 1.8 mg per square meter of body‐surface area per cycle; they received 0.8 mg on day 1 of each cycle and 0.5 mg on days 8 and 15. Cycle 1 lasted 21 days and the subsequent cycles each lasted 28 days; the patients received treatment for up to six cycles. Once a patient achieved complete remission or complete remission with incomplete hematologic recovery, the dose that was administered on day 1 of each cycle was reduced to 0.5 mg for the duration of the trial. Patients in the SoC group received the investigator's choice of one of the following three regimens: FLAG (fludarabine, cytarabine, and granulocyte colony‐stimulating factor) therapy for up to four 28‐day cycles, cytarabine plus mitoxantrone for up to four 15‐to‐20‐day cycles, or high‐dose cytarabine for up to two 12‐dose cycles. Details are provided in the respective trial publication.^16^ Admission for drug administration was neither recommended nor mandated, but was entirely based on physician judgment and local SoC.

All analyses presented in this paper were based on the January 2017 data cut of the INO‐VATE ALL trial and consider the safety population in the INO‐VATE ALL trial, which included all R/R ALL patients who were randomized to either INO or SoC therapy and received at least one dose of the respective therapy. With regard to comparison of hospitalization burden between treatments, number of patients with at least one hospitalization and the total number of days a patient was hospitalized from randomization until end of study treatment was calculated. Hospitalization days before randomization in the trial were excluded from this comparison, hospitalizations after end of study treatment were not documented in the INO‐VATE ALL trial.

Due to different durations of observation for INO and SoC, number of hospital days of all patients were mainly reported per observed patient month. Number of patients with at least one hospitalization and hospital days per patient month were also analyzed for different treatment cycles as well as with regard to main reasons for hospitalizations as documented by study physicians.

Number of hospitalization days per patient month was compared between all patients who received either INO or SoC by calculating the incidence rate ratio (IRR). The comparison was repeated within several subgroups: males vs females, R/R ALL patients treated in different regions of the world, older vs younger patients (<55 years vs >55 years), short vs long duration of first remission (<12 months vs >12 months), salvage status (first vs second), and Philadelphia status (positive vs negative).

Finally, in a multivariable analysis influence of the following independent variables on hospitalization burden, defined as hospital days per observed patient month, was estimated: age, gender, salvage status, Philadelphia status, duration of first remission, INO vs SoC treatment. Generally, due to the categorical nature of the dependent variable, either a negative binominal regression or a Poisson regression model was considered for this analysis.^21^, ^22^, ^23^ In order to identify the most suitable model, first, the requirement of overdispersion was tested. Afterward, fit of both types of models was assessed based on the Akaike's information criterion (AIC) as well as the Bayesian information criterion (BIC). The model with the best fit was chosen. To express coefficients of that model in a more interpretable form, we estimated the IRR related to observed hospitalization days per patient month for each independent variable in addition to the respective coefficients. In these estimates, the IRR can be interpreted as the ratio of two the number of hospitalization days per month in the INO vs the SoC arm.

All analyses were done with R 3.4.3 and Stata version 14.1 software. The INO‐VATE ALL trial was approved by an ethics commission,^16^ no separate approval for this analysis was obtained or deemed necessary. Some of the clinical data presented within this article has previously been presented at conferences.^24^, ^25^

## 3 RESULTS

In the INO‐VATE ALL trial (safety population), 164 patients received INO and 143 received SoC (93 patients received FLAG, 33 Ara‐C/Mito and 17 HIDAC). Mean age of the whole population was 46 years, 40.4% of patients were female, and about 48.5% of patients were treated at North American study sites. 66.8% received their salvage 1 therapy at date of study start, the remaining patients received salvage 2 therapy. Patients in the INO arm received a median of 3 cycles of study therapy compared to a median of 1 cycle for the SoC arm (Table 1). The majority of patients (85.7%) were Philadelphia negative. During the treatment period, patients in the INO arm were observed significantly longer than SoC patients (mean: 85.0 days vs 45.2 days per patient).

**Table 1:** Baseline characteristics of study population^a^

Characteristics	INO (N = 164)		SoC (N = 143)		Total (N = 307)	
	N	%	N	%	N	%
All patients	164	100.0	143	100.0	307	100.0
Observation days (mean/median)	85.0/77		45.2/37		66.5/50	
Number of treatment cycles started per patient (mean/median)	2.8/3.0		1.2/1.0		2.1/2.0	
Gender						
Male	91	55.5	92	64.3	183	59.6
Female	73	44.5	51	35.7	124	40.4
Age at randomization						
Mean/median age	45.9/47		45.6/47		45.7/47	
<55 years	104	63.4	91	63.6	195	63.5
≥55 years	60	36.6	52	36.4	112	36.5
Region						
North America	75	45.7	74	51.7	149	48.5
Europe	61	37.2	54	37.8	115	37.5
Asia	26	15.9	15	10.5	41	13.4
Australia	2	1.2	0	0.0	2	0.7
Duration of first remission at randomization						
<12 months	109	66.5	94	65.7	203	66.1
≥12 months	55	33.5	49	34.3	104	33.9
Salvage status at randomization						
Salvage 1	108	65.9	97	67.8	205	66.8
Salvage 2	56	34.1	46	32.2	102	33.2
Philadelphia status						
Philadelphia positive	22	13.4	22	15.4	44	14.3
Philadelphia negative	142	86.6	121	84.6	263	85.7

Abbreviations: INO, inotuzumab ozogamicin; SoC, standard of care.

a Baseline characteristics were based on IVRS classification and are consistent with the latest CSR data (January 2017). Therefore, they might be slightly different from previously published data. The describes characteristics of patients in both treatment arms as well as characteristics of the whole observed population. Patients are the “safety population” of the INO‐VATE ALL trial, defined as all patients who received at least one dosage of study medication.

Table 2 describes observed overall hospitalizations for trial patients as well as hospitalizations per treatment cycle. Overall, 136 of 164 (82.9%) INO patients and 135 of 143 (94.4%) SoC patients experienced at least one hospitalization. The mean hospitalization days per patient month in the INO arm was 7.6 days compared with 18.4 days in the SoC arm, resulting in an IRR of 0.413 (*P* < .001). During treatment cycle 1, the mean hospitalization days per patient month are 12.5 days in the INO arm and 18.9 days in the SoC arm (IRR: 0.661, *P* < .001). In cycles 2, 3, and 4 the hospitalizations in the INO arm are lower compared to the SoC arm; 6.1, 5.2, and 3.8 days per patient month vs 16.7, 14.1, and 8.8 days per patient month, respectively. The IRRs for cycles 2, 3, and 4 are 0.363 (*P* < .001), 0.365 (*P* < .001), and 0.424 (*P* = .0246).

**Table 2:** Hospitalizations in trial patients per treatment cycle^a^

	INO (N = 164; total number of observed patient days = 13 946)			SoC (N = 143; total number of observed patient days = 6463)			IRR related to hospital days per patient month INO vs SoC	*P*‐value
	Patients with at least one hospitalization (%)	Total number of hospitalization days	Hospitalization days per patient month	Patients with at least one hospitalization (%)	Total number of hospitalization days	Hospitalization days per patient month		
**Any hospitalization in the observation period (all cycles)**	**136 (82.9%)**	**3486**	**7.61**	**135 (94.4%)**	**3912**	**18.42**	**0.413**	**<.001**
Hospitalizations during treatment cycle 1 (nb. of patients who started cycle 1^b^: INO = 164/SoC = 143)	124 (75.6%)	1887	12.47	135 (94.4%)	3356	18.87	0.661	<.001
Hospitalizations during treatment cycle 2 (nb. of patients who started cycle 2^b^: INO = 127/SoC = 27)	54 (42.5%)	728	6.07	25 (92.6%)	475	16.73	0.363	<.001
Hospitalizations during treatment cycle 3 (nb. of patients who started cycle 3^b^: INO = 87/SoC = 5)	33 (37.9%)	473	5.17	5 (100.0%)	72	14.14	0.365	<.001
Hospitalizations during treatment cycle 4 (nb. of patients who started cycle 4 b: INO = 45/SoC = 1)	14 (31.1%)	184	3.75	1 (100.0%)	9	8.84	0.424	.0246
Hospitalizations during treatment cycle 5 (nb. of patients who started cycle 5^b^: INO = 26/SoC = 0)	9 (34.6%)	150	5.40	—	—	—	—	—
Hospitalizations during treatment cycle 6 (nb. of patients who started cycle 6^b^: INO = 15/SoC = 0)	3 (20.0%)	64	3.48	—	—	—	—	—

Note

The comparison of observed hospital days per patient month for the overall observation period (all cycles) and for the different cycle periods. Comparison was done using IRR statistics.

The bold values indicates hospitalization in all cycles (which is the sum of each of the individual cycles below).

Abbreviations: INO, Inotuzumab ozogamicin; IRR, Incidence rate ratio; SoC, Standard of care.

a Data analyses were performed based on individual patient‐level data from the January 2017 data cut, which was consistent with the January 2017 data cut.

b Used as denominator for percentage calculations.

**Figure 1:**
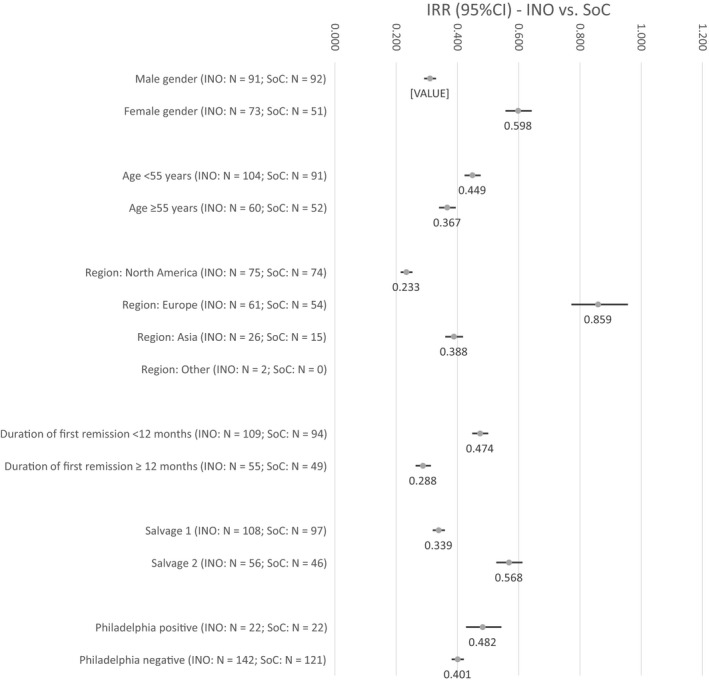
Incidence rate ratio (95% CI) comparing hospitalized days per patient month between INO and SoC in different pre‐defined subgroups

Based on the overall observational period, Figure 1 describes the IRRs comparing the hospitalization days per patient month in the INO arm with the SoC arm within pre‐defined subgroups. In all comparisons, hospital burden, defined as observed hospitalization days per patient month, was lower in the INO arm compared with the SoC arm. Highest differences could be observed in the subgroup of patients included in North America with an IRR_(INO vs. SoC)_ of 0.233 (*P* < .001) and in patients with a duration of first remission at randomization of ≥12 months with an IRR_(INO vs. SoC)_ of 0.288 (*P* < .001). Lowest difference between INO vs SoC regarding the hospitalized days per patient months was identified in the European patient subgroup (IRR_(INO vs. SoC)_: 0.859, *P* < .001).

Table 3 outlines the main reasons for the documented hospitalizations in the INO arm and the SoC arm. It reports both number of patients with at least one hospitalization related to the respective reason and the number hospital days per observed patient month per documented reason. Most of hospitalizations (56.7% in INO arm (93/164 patients) and 81.8% in SoC arm (117/143 patients) with at least one hospitalization) were associated with R/R ALL treatment. The number of hospital days per observed patient month related to R/R ALL treatment was significantly lower in the INO vs SoC arm (5.2 days vs 14.0 days, IRR = 0.368, *P* < .001). 28.7% of the patients in the INO arm experienced at least one hospitalization related to toxicities of treatment, whereas it was 21.0% of the patients in the SoC arm. However, INO patients spent a substantially longer time on treatment (in total 13 946 days [85 days per patient] vs 6463 days [45 days per patient]). The resulting number of hospitalization days per patient month on treatment because of treatment toxicities were thus lower for INO in comparison to SoC (1.4 vs 2.8 days, IRR = 0.516, *P* < .001). Finally, 23.2% (INO) vs 12.6% (SoC) of all patients had at least one documented hospitalization associated for other reasons or non‐specified illnesses. INO patients experienced 1.0 hospital days per observed patient month because of these reasons, whereas it was 1.6 days per patient month for SoC patients (IRR = 0.629, *P* < .001).

**Table 3:** Documented reasons for hospitalization events and observed hospitalization days, during all observed treatment cycles

	INO (N = 164; total number of observed patient days = 13 946)			SoC (N = 143; total number of observed patient days = 6463)			IRR related to hospital days per patient month INO vs SoC	*P*‐value
	Patients with at least one hospitalization due to the respective reason [n (%)]	Total number of hospitalization days	Hospitalization days per patient month	Patients with at least one hospitalization due to the respective reason [n (%)]	Total number of hospitalization days	Hospitalization days per patient month		
**Treatment of underlying disease including preparation for HSCT**	**93 (56.7)**	**2359**	**5.15**	**117 (81.8)**	**2970**	**13.98**	**0.368**	**<.001**
Scheduled treatment related to ALL / study related	75 (45.7)	1883	4.11	91 (63.6)	2256	10.62	0.387	<.001
Relapse	18 (11.0)	380	0.83	25 (17.5)	676	3.18	0.261	<.001
Preparation for HSCT	6 (3.7)	96	0.21	1 (0.7)	38	0.18	1.171	.414
**Treatment toxicities**	**47 (28.7)**	**662**	**1.44**	**30 (21.0)**	**595**	**2.80**	**0.516**	**<.001**
Infection	17 (10.4)	245	0.53	8 (5.6)	205	0.96	0.554	<.001
Neutropenia	1 (0.6)	3	0.01	1 (0.7)	30	0.14	0.046	<.001
Neutropenic fever	16 (9.8)	198	0.43	15 (10.5)	202	0.95	0.454	<.001
Other cytopenias	1 (0.6)	17	0.04	1 (0.7)	4	0.02	1.970	.220
Organ dysfunction (renal, pulmonary, cardio, neuro, liver, GI, skin etc)	10 (6.1)	140	0.31	2 (1.4)	62	0.29	1.046	.773
Bleeding (all sites)	5 (3.0)	33	0.07	0 (0.0)	0	0.00	—	<.001
Tumor lysis	1 (0.6)	2	0.00	0 (0.0)	0	0.00	—	.447
Sepsis	3 (1.8)	24	0.05	7 (4.9)	92	0.43	0.121	<.001
**General or non‐specific illness**	**38 (23.2)**	**471**	**1.03**	**18 (12.6)**	**347**	**1.63**	**0.629**	**<.001**
Transfusion	2 (1.2)	3	0.01	2 (1.4)	38	0.18	**0.037**	<.001
Surgery	1 (0.6)	7	0.02	0 (0)	0	0.00	—	.070
Fall	1 (0.6)	18	0.04	0 (0)	0	0.00	—	.001
Non‐specific symptoms	22 (13.4)	220	0.48	8 (5.6)	96	0.45	1.062	.627
Mixed	16 (9.8)	223	0.49	8 (5.6)	213	1.00	0.485	<.001

Note

The number of hospitalizations as well as number of observed hospital days, per hospitalization reason as documented by the study physicians (determined based on individual patient‐level data from the January 2017 data cut). Observed days always report to the whole observational period. The IRR (incidence rate ratio) was calculated comparing observed hospital days per patient month, statistical comparisons were done using IRR statistics.

The bold values indicates the three main categories of hospitalization reasons (which are the sum of each of the individual reasons below).

Abbreviations: ALL, acute lymphoblastic leukemia; GI, gastro‐intestinal; HSCT, hematopoietic stem cell transplantation; INO, inotuzumab ozogamicin; IRR, incidence rate ratio; SoC, standard of care.

Table 4 summarizes the results of the multivariable analysis of factors being associated with the number of hospitalization days per observed patient month. In this model, age, gender, a duration of first remission of <12 months, salvage stage as well as Philadelphia status were not significantly associated with the dependent variable. However, patients having been treated in Asia experienced a higher hospitalization burden (IRR = 2.210 compared with North America, *P* < .001). Finally, INO was associated with a significantly lower number of hospital days per patient month, in comparison to SoC (IRR = 0.404, *P* < .001).

**Table 4:** Results of a multivariable analysis of factors associated with the number of hospitalization days per patient month

	Coefficient	Standard error	IRR (95% CI)	*P*‐value
Treatment				
SoC	Reference			
INO	−0.907	0.106	0.404 (0.328‐0.497)	<.001
Gender				
Male	Reference			
Female	−0.151	0.107	0.860 (0.697‐1.061)	.160
Age (years)	0.0001	0.003	1.000 (0.994‐1.006)	.962
Region				
North America	Reference			
Asia	0.793	0.166	2.210 (1.597‐3.060)	<.001
Europe	0.129	0.113	1.138 (0.912‐1.419)	.252
Other	0.313	0.653	1.367 (0.380‐4.915)	.632
Duration of first remission at randomization				
≥12 months	Reference			
<12 months	0.173	0.110	1.189 (0.958‐1.476)	.116
Salvage status at randomization				
Salvage 1	Reference			
Salvage 2	0.128	0.112	1.136 (0.912‐1.416)	.256
Philadelphia status				
Philadelphia negative	Reference			
Philadelphia positive	0.071	0.151	1.073 (0.799‐1.442)	.639
Constant	2.656	0.231		<.001
Alpha	0.717	0.071		.272

Note

Negative binomial regression‐Number of observations = 307; LR χ^2^(9) = 81.05; Dispersion = mean; Prob>χ^2^<0.001; Log likelihood = −1102.59; Pseudo *R* ^2^ = 0.0355. Reports results of the multivariable binomial regression analysis of factors being associated with the observed number of hospital days per patient month, per patient. For this analysis, a negative binomial regression model was used, as the likelihood ratio test of the overdispersion parameter alpha showed that alpha was significantly different from zero and thus, indicated that the Poisson distribution is not appropriate. Furthermore, the negative binomial model showed a better fit (AIC = 2227.20; BIC = 2268.19) than the Poisson regression model (AIC = 3450.43; BIC = 3487.70).

Abbreviations: INO, inotuzumab ozogamicin; IRR, incidence rate ratio; SoC, standard of care.

## 4 DISCUSSION

The INO‐VATE ALL trial showed patients receiving INO vs SOC patients achieved higher response (80.7% vs 29.4%, *P* < .001), MRD‐negativity rates (2.8‐fold increase; *P *< .001), and prolonged progression‐free survival and overall survival. Veno‐occlusive disease (VOD) was a major non‐hematologic toxicity.^16^ Using the documented on‐treatment hospitalizations of the INO‐VATE ALL trial, the main aim of our analysis was to report treatment‐associated hospital burden of R/R ALL patients and to compare this burden between patients who received either a treatment with INO or SoC.

We confirm the results of earlier studies that hospital burden is high in this patient population.^17^, ^18^, ^19^, ^20^ On average, patients observed in our study experienced a mean of 11.0 hospital days per observed patient month (INO 7.6 days, SoC 18.4 days), which translates into 36.2% of time that patients were hospitalized (INO: 25.0% of observed time, SoC: 60.5% of observed time). Compared to previous observations, that reported hospital burden associated with chemotherapy only, SoC patients in our study spent a slightly higher proportion of their treatment time in hospitals. A Belgium study reported that Philadelphia negative R/R ALL patients spent about 50% of their time during salvage chemotherapy in the hospital.^19^ Similarly, a US claims data study on Philadelphia‐negative R/R ALL patients concluded that patients stayed 56% of the observed time since first R/R ALL diagnosis in the hospital,^17^ whereas a French chart review reported 46% and a Spanish chart review reported 53% in this respect^18^, ^20^. This finding is in line with our expectations as these studies are based on real‐world evidence, whereas our findings are based on clinical trial data, where the probability of being hospitalized might be higher.

However, INO treatment was still associated with a substantially lower hospital burden as INO patients spent only 25.0% of their treatment time in a hospital. This was associated with a lower percentage of INO patients with at least one hospitalization and a shorter duration of hospitalizations in the INO arm.

A number of reasons might explain this finding. First, as shown in the R/R ALL trial, INO is associated with a higher remission rate. This might translate into less frequent and shorter hospitalizations in association with ALL treatment. Additionally, INO is administered in a one‐hour weekly dosing schedule that does not require a hospitalization for drug administration purposes. As a result, INO patients stayed only 5.2 days per patient month in the hospital because of R/R ALL treatment (including preparation for SCT), whereas SoC patients stayed 14.0 days per patient month in hospital because of this reason (Table 3). Second, INO treatment was also associated with lower safety‐related hospitalizations (1.4 days INO vs 2.8 days SoC per patient month resulting in an IRR of 0.516; *P* < .001), even though the reported SAE rate was similar between both treatments (46% SAE rate for INO patients vs 43% for SoC patients).^16^ Finally, hospitalizations due to other reasons (1.0 days INO vs 1.6 days SoC per patient month resulting in an IRR of 0.629; *P* < .001) were lower in the INO treatment arm as well. The precise reasons for this difference are unknown. Above main result of a lower hospital burden associated with INO treatment were confirmed in all subgroup analyses.

We acknowledge some limitations of our analysis. First, we only compared INO with SoC chemotherapy, as in the INO‐VATE trial. Therefore, other treatments such as blinatumomab were not included in our comparison. Future comparisons between INO and other treatment options are needed as well. Second, our analyses were based on the INO‐VATE ALL trial. As this was a randomized open‐label trial, we believe that our comparison of hospital burden between INO and SoC patients has been done with a good internal reliability. However, the INO‐VATE ALL trial may not reflect everyday clinical practice, as both treatment and follow‐up observation of patients were highly influenced by the respective clinical study protocol. In line with that, hospital burden was higher in our SoC arm than in previous observational studies. So, in the real‐world, we expect a lower hospital burden than that reported in our study, with similar or improved relative effects of INO vs SoC treatment, as INO can be administered in the outpatient setting freely whereas in the INO‐VATE ALL trial there might have been a tendency towards an earlier hospitalization of a patient.^26^ Finally, as hospitalizations were documented on treatment only in the R/R ALL trial, our analysis only refers to these periods. This implies that our current analysis does not capture the potential impact of INO‐related VOD on hospitalizations, as VOD occurs within the HSCT period and not during treatment with either INO or SoC. Whether there are other differences in hospitalization burden between INO and SoC patients after end of treatment is unknown.

We conclude that INO treatment is not only more effective with a significantly greater CR/CRi rate than SoC in R/R ALL patients but is also associated with lower hospital burden. In line with this, in a previous analysis of the INO‐VATE ALL trial data, Kantarjian et al found that, regardless of the treatment received, non‐hospitalized patients exhibited improvements in measured patient‐reported outcomes such as the European Organization for Research and Treatment of Cancer (EORTC) Quality of Life Questionnaire (EORTCQLQ‐C30) and the EuroQol Group 5 Dimensions Questionnaire (EQ‐5D), whereas hospitalized patients exhibited deteriorations or minimal changes from baseline in this respect.^27^ Therefore, it is expected that the lower hospital burden of INO is likely to contribute to better patient reported outcomes, with patients being more ambulatory and realizing significantly less negative impact on their daily lives. Even if INO patients are generally treated longer and a higher percentage proceeds to SCT, we also conclude that the above lower hospital burden probably has a favorable impact on health care budgets and cost‐effectiveness considerations. Collection of further data in the real‐world setting is warranted to verify and further quantitate these findings.

## CONFLICTS OF INTEREST

This study was sponsored by Pfizer Inc David I. Marks and Hagop Kantarjian were part of the advisory board and received honoria from Pfizer. Ilse van Oostrum and Sabrina Mueller are employees of Ingress‐Health, who were paid consultants to Pfizer in connection with the development of this manuscript. Verna Welch, Erik Vandendries, Fausto R. Loberiza, and Sarah Böhme are employees at Pfizer. Yun Su is a former employee at Pfizer. Matthias Stelljes reports research support from Pfizer during the conduct of the study and other consulting fees and honoraria from Pfizer, Amgen, and Jazz Pharmaceuticals.

## AUTHOR CONTRIBUTIONS

David I. Marks: Study design, data analysis, contributed to writing of manuscript, approval. Ilse van Oostrum: Approval, Ilse van Oostrum has conducted parts of the data analysis and drafted and updated the manuscript. Sabrina Mueller: Approval, SM has conducted parts of the analysis, drafted parts of the methods/results sections and supported the interpretation of data. Verna Welch: contributed to the analysis and interpretation of data; revised draft versions of the manuscript; and gave final approval. Erik Vandendries: contributed to the analysis and interpretation of data; revised draft versions of the manuscript; and gave final approval. Fausto R. Loberiza: contributed to the analysis and interpretation of data; revised draft versions of the manuscript; and gave final approval. Sarah Bohme: Approval, SB contributed to the development of the research question and has been involved in the planning of the analyses. Further, SB contributed to the final version of the manuscript. Yun Su: contributed to the analysis and interpretation of data; revised draft versions of the manuscript; and gave final approval. Matthias Stelljes: contributed to the analysis and interpretation of data; revised draft versions of the manuscript; and gave final approval. Hagop M. Kantarjian: Study design, data analysis, contributed to writing of manuscript, approval.
